# Meteorological Register

**Published:** 1814-06

**Authors:** C. Blunt

**Affiliations:** Philosophical Instrument Maker, No. 38, Tavistock-Street, Covent-Garden


### METEOROLOGICAL REGISTER.

*From April the 25th, to May the 25th,* 1814.

Kept by C. BLUNT, Philosophical Instrument Maker, No. 38, Tavistock-Street, Covent-Garden.

| Moon. | Day. | Wind. | Barometrical Pressure. Max. | Min. | Mean. | Temperature. Max. | Min. | Mean. | |
|---|---|---|---|---|---|---|---|---|---|
| ◑ | 26 | SE | 30·05 | 29·89 | 29·975 | 56 | 36 | 43 | Showers |
| | 27 | SE | 30.13 | 30·11 | 30·123 | 51 | 46 | 48·6 | Showers |
| | 28 | S | 30·13 | 30·13 | 30·13 | 54 | 48 | 51 | Rain |
| | 29 | S | 30·07 | 30·03 | 30·056 | 59 | 48 | 53·6 | Fair |
| | 30 | S | 30·10 | 30·04 | 30·07 | 65 | 48 | 56·6 | Fair |
| | 1 | S | 30·14 | 30·11 | 30·13 | 69 | 50 | 58·6 | Fair |
| | 2 | E | 30·16 | 30·12 | 30·146 | 68 | 46 | 56·3 | Clear |
| | 3 | SE | 30·02 | 29·90 | 29·95 | 64 | 41 | 54 | Clear |
| ○ | 4 | SE | 29·83 | 29·75 | 29·783 | 59 | 38 | 49·3 | Clear |
| | 5 | E | 29·53 | 29·18 | 29·366 | 52 | 40 | 45·6 | Rain |
| | 6 | NW | 29·36 | 29·16 | 29·253 | 61 | 44 | 53·6 | Showery |
| | 7 | E | 29·75 | 29·57 | 29·653 | 59 | 39 | 50 | Fair |
| | 8 | E | 29·96 | 29·75 | 29·83 | 58 | 37 | 47·3 | Cloudy |
| | 9 | E | 30·19 | 30·01 | 30·093 | 59 | 39 | 47 | Cloudy |
| | 10 | NE | 30·35 | 30·27 | 30·30 | 56 | 40 | 46·6 | Cloudy |
| | 11 | E | 30·45 | 30·34 | 30·396 | 54 | 42 | 46·3 | Cloudy |
| ◐ | 12 | NE | 30·29 | 30·20 | 30·243 | 53 | 37 | 45·3 | Rain |
| | 13 | NE | 30·10 | 30·— | 30·043 | 54 | 40 | 45 | Rain |
| | 14 | NE | 29·98 | 29·97 | 29·976 | 55 | 37 | 45·6 | Fair |
| | 15 | NE | 30·— | 29·54 | 30·076 | 58 | 39 | 48 | Fair |
| | 16 | SE | 30·01 | 29·95 | 29·986 | 58 | 44 | 51·6 | Fair |
| | 17 | SE | 30·15 | 30·11 | 30·123 | 59 | 46 | 52 | Fair |
| | 18 | SE | 30·18 | 30·09 | 30·13 | 60 | 47 | 54·3 | Fair |
| | 19 | E | 30·09 | 30·05 | 30·066 | 58 | 45 | 52 | Fair |
| ● | 20 | E | 30·— | 29·94 | 29·966 | 60 | 44 | 51·6 | Fair |
| | 21 | E | 29·90 | 29·82 | 29·87 | 59 | 42 | 49·6 | Boisterous Wind |
| | 22 | NE | 29·96 | 29·72 | 29·846 | 55 | 37 | 47 | Fair |
| | 23 | N | 29·66 | 29·50 | 29·553 | 56 | 39 | 47 | Fair |
| | 24 | N | 29·57 | 29·56 | 29·563 | 54 | 38 | 45·3 | Rain & stormy Wind |
| | 25 | NE | 29·76 | 29·67 | 29·723 | 55 | 35 | 45 | Rain & stormy Wind |

RESULTS.

Mean barometrical pressure 29·934
Maximum 30·45 inc. wind at E
Minimum 29·16 ——— NW

Mean temperature 49·4 deg.

Maximum 69, the wind at S

Minimum 35, ——— NE

*Scale exhibiting the prevailing Winds during the Month.*

| N | NE | E | SE | S | SW | W | NW |
|---|---|---|---|---|---|---|---|
| 2 | 7 | 9 | 7 | 4 | 0 | 0 | 1 |

| | Mean barometrical pressure. | Mean temperature. |
|---|---|---|
| From the first quarter on the 26th April, to the full moon on the 4th May | 30·072 | 52·7 |
| ——— full moon, to the last quarter on the 12th | 29·834 | 48·2 |
| ——— last quarter, to the new moon on the 19th | 30·080 | 49·2 |
| ——— new moon, to the first quarter on the 26th | 29·753 | 47·5 |

*Observations.*—There is little to notice in the Register of the Month but but the unusual low state of temperature for the advanced state of the season.

In order to produce as distinct views as possible of the meteorology of the period, the present Register has two additional columns, one shewing the mean barometrical pressure of each day, the next its mean temperature: the mean temperature and pressure during each *lunar division* of the month, and the mean pressure and temperature of the whole period, are found, as usual, below the Register.

It is necessary to explain, that, although the columns of the Register exhibit only the extremes of pressure and temperature for each day, the columns of the mean pressure and temperature, the mean pressure and temperature of the lunar divisions, and of the whole month, are deduced from *four* daily observations on each instrument, viz. of the barometer at 12 P. M., 8 A. M, noon, and 8 P. M.; and of the thermometer at 12 P. M. The minimum by a night-registering thermometer between midnight and 8 A. M.; at 8 the maximum of that day also by a self-registering thermometer, and at 8 P. M.

This state of weather easily accounts for the number and severity of catarrhal and pulmonary affections which still claim our constant care. Measles, hooping cough, and scarlet fever, have appeared in several families, with their usual symptoms. In a case of porrigo, the subject of it a girl between seven and eight years old, menstruated at the age of six years and a half, the menstrual discharge continued the usual time, and according to the mother's report resembled in every particular that of women. The child has since been subject to an occasional discharge from the vagina resembling that of leucorrhœa; but the catamenia have not recurred. She is not more forward in disposition, or premature in appearance, than other children of her age; and the organs of generation are not more advanced than usual, neither do they appear to have suffered from any external injury.

